# Enhanced efficiency in mitotane therapeutic drug monitoring: optimization of chromatographic resolution and column longevity

**DOI:** 10.1016/j.clinsp.2026.101069

**Published:** 2026-07-29

**Authors:** Anna Sylvia Ferrari Marques, Berenice Bilharinho Mendonca, Helena Panteliou Lima-Valassi

**Affiliations:** Núcleo Multiusuário de Cromatografia Líquida Associada à Espectrometria de Massas em Tandem, (AE-06 Rede Premium); Laboratório de Hormônios e Genética Molecular LIM-42, Hospital das Clínicas da Faculdade de Medicina da Universidade de São Paulo, São Paulo, SP, Brazil

Dear Editor,

This investigation aims to address the analytical challenges associated with mitotane [2,4′DDD; 1-(2-chlorophenyl)-1-(4-chlorophenyl)-2,2-dichloroethane] therapeutic drug monitoring by presenting an updated Liquid Chromatography-Diode Array Detection (LC-DAD) method, following the recent developments reported by Marques et a.^1^ in this journal. While the current internal standard, 4,4-DDD [1,1-dichloro-2,2-bis(4-chlorophenyl)ethane], demonstrated effective performance, the continuous drive for method improvement and adaptation to new analytical requirements prompted the consideration for changing both the internal standard and the chromatographic column. The previous internal standard proved difficult to maintain a robust separation in the chromatography column used, an Acquity Waters HSS T3 (100 × 2.1 mm, 1.8 µm); due to structural similarity, baseline separation was difficult to achieve during continuous column usage, leading to poor resolution and potentially erroneous results. Furthermore, the column showed poor robustness, high background pressure, and frequent blocking.

The proposed new internal standard, 2,4’-DDT [1,1,1-trichloro-2-(2-chlorophenyl)-2-(4chlorophenyl)ethane], exhibited the required similarity in extraction recovery, matrix effect, and chromatographic behavior to mitotane, but with superior performance. The 2,4’-DDT co-extracted well with mitotane and, crucially, was better separated from the analyte and other endogenous plasma components. Another significant improvement was the decision to switch to a Phenomenex Kinetex C18 (150 × 2.1 mm, 100 Å) column, which offered substantial improvements in speed and efficiency, aligning with the goal of a simplified and rapid quantitative method for mitotane monitoring. However, this transition necessitated careful consideration and re-optimization due to inherent differences in bonding chemistry, endcapping, and silica properties between manufacturers. These differences can lead to subtle but significant changes in selectivity, meaning the retention times and elution order of mitotane and co-eluting matrix components might shift. Consequently, the change in column technology required a re-optimization of chromatographic conditions, including flow rate and column temperature, while the mobile phase composition and pH were kept the same.

This was crucial to achieve superior separation of mitotane from potential interferences and the new proposed internal standard.^2^ A partial re-validation of the method was performed, including assessing specificity, linearity, precision, accuracy, recovery, and robustness.^3^^,^^4^^,^^7^ This ensures that the new proposed column maintains or even improves the method's reliability and suitability for clinical application. In summary, transitioning to a Phenomenex Kinetex C18 column and using 2,4´DDT as the internal standard offered enhanced speed, robustness, and efficiency.^5^ This comprehensive re-evaluation ensures that the updated LC-DAD method achieves a high level of precision and minimizes experimental error, which is crucial for reliable clinical application.^6^ The selectivity of the method was confirmed by observing distinct chromatographic peaks for mitotane with appropriate retention times, demonstrating minimal interference from endogenous compounds or other co-administered medications.^8^

The limit of quantification was set at a level ensuring reliable detection for therapeutic monitoring, aligning with established guidelines.^9^ The lower and upper limits of quantification were kept the same (0.25 µg/mL to 50.0 µg/mL) to define the working range across the clinically relevant spectrum. Moreover, the recovery rate for mitotane was consistently high, above 80%, indicating efficient extraction from plasma without significant loss, while matrix effects were investigated to ensure that biological components did not interfere with quantification. Specificity was rigorously assessed to confirm the method's ability to quantify mitotane in the presence of metabolites and endogenous constituents. The method's linearity demonstrated strong correlation coefficients (*r* > 0.99), underscoring its capability over the entire therapeutic range. Precision, assessed through intra-day and inter-day variability, consistently fell within acceptable limits, confirming reproducibility for routine clinical use.^7^ Accuracy, evaluated by comparison with the previously developed method.^1^ and spiked samples, demonstrated excellent agreement with nominal concentrations. In conclusion, the updated LC-DAD method for mitotane plasma quantification demonstrates excellent analytical performance across all validation parameters, making it a reliable and accessible option for therapeutic drug monitoring.^10^

## Data availability statement

Not applicable.

## Declaration of competing interest

Helena Panteliou Lima Valassi reports financial support was provided by Clinical Hospital of the Faculty of Medicine of the University of São Paulo. Anna Sylvia Ferrari Marques reports financial support was provided by Clinical Hospital of the Faculty of Medicine of the University of São Paulo. Berenice Bilharinho Mendonca reports financial support was provided by Clinical Hospital of the Faculty of Medicine of the University of São Paulo. If there are other authors, they declare that they have no known competing financial interests or personal relationships that could have appeared to influence the work reported in this paper.
